# Status of Digital Health Technology Adoption in 5 Vietnamese Hospitals: Cross-Sectional Assessment

**DOI:** 10.2196/53483

**Published:** 2025-02-06

**Authors:** Duc Minh Tran, Nguyen Thanh Dung, Chau Minh Duc, Huynh Ngoc Hon, Le Minh Khoi, Nguyen Phuc Hau, Duong Thi Thu Huyen, Huynh Thi Le Thu, Tran Van Duc, VITAL (Vietnam ICU Translational Applications Laboratory) Investigators, Lam Minh Yen, C Louise Thwaites, Chris Paton

**Affiliations:** 1 Oxford University Clinical Research Unit Ho Chi Minh City Vietnam; 2 Hospital for Tropical Diseases Ho Chi Minh City Vietnam; 3 Dong Thap General Hospital Dong Thap Vietnam; 4 Trung Vuong Hospital Ho Chi Minh City Vietnam; 5 University Medical Center Ho Chi Minh City Vietnam; 6 Department of Information Technology National Hospital for Tropical Diseases Hanoi Vietnam; 7 See Acknowledgements; 8 Centre for Tropical Medicine and Global Health University of Oxford Oxford United Kingdom; 9 Nuffield Department of Medicine University of Oxford Oxford United Kingdom; 10 Department of Information Science University of Otago Dunedin New Zealand

**Keywords:** electronic health record, electronic medical record, digital maturity, clinical decision support, digital infrastructure, Vietnam, health information technology, digital health technology, low- and middle-income country

## Abstract

**Background:**

Digital health technologies (DHTs) have been recognized as a key solution to help countries, especially those in the low- and middle-income group, to achieve the Sustainable Development Goals (SDGs) and the World Health Organization’s (WHO) Triple Billion Targets. In hospital settings, DHTs need to be designed and implemented, considering the local context, to achieve usability and sustainability. As projects such as the Vietnam ICU Translational Applications Laboratory are seeking to integrate new digital technologies in the Vietnamese critical care settings, it is important to understand the current status of DHT adoption in Vietnamese hospitals.

**Objective:**

We aimed to explore the current digital maturity in 5 Vietnamese public hospitals to understand their readiness in implementing new DHTs.

**Methods:**

We assessed the adoption of some key DHTs and infrastructure in 5 top-tier public hospitals in Vietnam using a questionnaire adapted from the Vietnam Health Information Technology (HIT) Maturity Model. The questionnaire was answered by the heads of the hospitals’ IT departments, with follow-up for clarifications and verifications on some answers. Descriptive statistics demonstrated on radar plots and tile graphs were used to visualize the data collected.

**Results:**

Hospital information systems (HIS), laboratory information systems (LIS), and radiology information systems–picture archiving and communication systems (RIS-PACS) were implemented in all 5 hospitals, albeit at varied digital maturity levels. At least 50% of the criteria for LIS in the Vietnam HIT Maturity Model were satisfied by the hospitals in the assessment. However, this threshold was only met by 80% and 60% of the hospitals with regard to HIS and RIS-PACS, respectively. Two hospitals were not using any electronic medical record (EMR) system or fulfilling any extra digital capability, such as implementing clinical data repositories (CDRs) and clinical decision support systems (CDSS). No hospital reported sharing clinical data with other organizations using Health Level Seven (HL7) standards, such as Continuity of Care Document (CCD) and Clinical Document Architecture (CDA), although 2 (40%) reported their systems adopted these standards. Of the 5 hospitals, 4 (80%) reported their RIS-PACS adopted the Digital Imaging and Communications in Medicine (DICOM) standard.

**Conclusions:**

The 5 major Vietnamese public hospitals in this assessment have widely adopted information systems, such as HIS, LIS, and RIS-PACS, to support administrative and clinical tasks. Although the adoption of EMR systems is less common, their implementation revolves around data collection, management, and access to clinical data. Secondary use of clinical data for decision support through the implementation of CDRs and CDSS is limited, posing a potential barrier to the integration of external DHTs into the existing systems. However, the wide adoption of international standards, such as HL7 and DICOM, is a facilitator for the adoption of new DHTs in these hospitals.

## Introduction

The COVID-19 pandemic demonstrated the need for countries worldwide to implement a national digital health infrastructure needed to respond to health emergencies and to achieve the Sustainable Development Goals (SDGs) [1]. As part of this digital transformation effort, digital health technologies (DHTs), such as electronic medical record (EMR) systems, laboratory information systems (LISs), and picture archiving and communication systems (PACSs), have become widespread. In recent years, Vietnam has developed and begun implementation of an ambitious national digital health strategy that includes the deployment of hospital-based EMRs and an electronic health insurance claim system [2].

As the cost of hardware continues to drop and the range and capabilities of DHTs continue to expand [3], digitization of health systems is becoming increasingly feasible in low- and middle-income countries (LMICs). New technologies, such as the Internet of Things, wearable devices, and artificial intelligence (AI), have begun to be adopted in health care settings and could be an important enabler for realizing the World Health Organization’s (WHO) Triple Billion Targets (more than 1 billion people benefiting from universal health care; better protected from health emergencies; and enjoying better health and well-being) and to accelerate SDG achievement [4]. In 2023, WHO announced the Global Initiative for Digital Health to help LMICs achieve the aims of the Global Strategy on Digital Health by developing their national digital health infrastructure, adopting international data standards, and fostering increased international collaboration in digital health [1,4].

In global health research, there is increasing interest in implementing new technologies, such as AI or smart wearables, for clinical decision support [5,6]. However, for these systems to be practicable and to take advantage of increasingly available “big data,” the local provision of technological infrastructure and implementation capabilities is necessary. For example, hospitals that have implemented EMR systems could use data from the EMRs to train AI models and enable the implementation of clinical decision support systems (CDSS) [7]. In addition, the adoption of international interoperability standards, such as Health Level Seven (HL7) Fast Healthcare Interoperability Resources (FHIR), is also necessary to allow efficient data exchange between information systems and new DHTs [8].

In hospital settings, digital maturity models, such as the Electronic Medical Record Adoption Model (EMRAM) from the Healthcare Information and Management Systems Society (HIMSS), are often used to guide DHT adoption and implementation [9]. These models can be used on a national or international scale and can target different dimensions, including technology, strategy, interoperability, analytics, and governance. Currently, there is no international consensus on how to best measure digital capabilities in health care institutions [9,10], and variations in digital maturity models across different countries may reflect an adaptation to national priorities and contexts, especially when being implemented in LMICs. In 2017, the Vietnam Ministry of Health (MoH) issued the Vietnam Health Information Technology (HIT) Maturity Model, originally known as Circular 54/2017/TT-BYT, as a roadmap for Vietnamese public hospitals toward a paperless and smart hospital model [2,11]. Although this guidance defines 7 digital maturity stages similar to EMRAM, the criteria to achieve a specific level have been significantly modified. For example, the Vietnam model includes the requirement for hospital information systems (HIS) to connect to the Vietnam Social Security claim portal and assesses the integration of the national standardized vocabularies into the digital systems [11].

The Vietnam ICU Translational Applications Laboratory (VITAL) project focuses on developing new technologies for improving outcomes of critically ill patients in Vietnam, such as CDSS using AI algorithms trained on local data [12-14]. In the national DHT policy landscape, regulations, infrastructure, and professional workflows are important elements [2,15] to ensure the sustainability and successful implementation of DHTs [6,16]. For this reason, in this study, we aimed to explore the current status and likely short-term development of the DHT infrastructure in 5 major public hospitals in Vietnam. Although the findings may not be generalizable to all Vietnamese hospitals regarding the state of their DHT adoption, we expect they can inform discussions around how new DHTs can be integrated with the existing IT infrastructure in Vietnamese public hospitals.

## Methods

### Study Design

This study used a structured questionnaire to explore the current adoption of DHTs in 5 public hospitals in Vietnam. The assessment items were developed based on the Vietnam HIT Maturity Model, which comprises 8 domains related to technology adoption and digital capabilities in hospitals [11]. The 8 domains are *IT infrastructure*, *HIS*, *LIS*, *radiology information systems–picture archiving and communication systems (RIS-PACS)*, *EMR*, *administrative and operation software*, *security and information safety*, and *nonfunctional criteria*. Except for security and information safety and nonfunctional criteria, each domain includes a set of infrastructure requirements, such as having a server room, or software functional criteria, such as having laboratory test management functionality. The model also sets out further digital capabilities focusing on assisting clinical practice, such as CDSS, clinical data repositories (CDRs), and medication management; these are named *extra capabilities*.

Following a stage-based approach, a hospital reaches a digital maturity level in a domain if a predefined set of criteria is met. The hospital can only move to a higher digital maturity level when all criteria of the lower levels have been satisfied. The domains of IT infrastructure and HIS have 7 digital maturity levels (1-7), while the other domains have 2 levels (*basic* and *advanced*). Multimedia Appendix 1 presents the digital maturity levels and their scoring criteria based on the domains and extra capabilities.

### Questionnaire, Data Collection, and Analysis

Since the focus of this study was the adoption of digital health systems in hospitals, we developed an assessment tool based on the criteria from the IT infrastructure, HIS, LIS, RIS-PACS, EMR, and extra capabilities domains (see Multimedia Appendix 2). The administrative and operation software, security and information safety, and nonfunctional criteria domains were not included in this study as we considered these less relevant to the study’s focus despite their importance to the overall digital maturity of a health care institution.

For each domain, we gathered information through the hospitals’ IT departments to determine whether the hospitals met the component criteria or whether they planned to achieve the criteria in the next 3-5 years. Results of the assessments were discussed with the heads of the IT departments to ensure accuracy. Data collection was carried out from July 2022 to March 2023. Information about the digital maturity of each hospital was entered into Microsoft Excel for initial data cleaning and then analyzed using R software (R Foundation for Statistical Computing) [16].

Our aim was to provide a descriptive summary of what infrastructure and functionalities, or criteria, were available in each hospital across the domains rather than calculating scores for their digital maturity. The proportion of criteria that each hospital met in each domain was calculated. A radar plot was created using the *fmsb* package in R [17] to depict the difference in the proportions of criteria met among the hospitals by domain. Radar plots are regarded as an efficient tool to compare various groups on multiple variables, as discussed by Saary [18]. In addition to summary statistics, we further compared technology adoption between the 5hospitals by delving into the individual domains. Tile graphs were created using the *ggplot2* package in R [19] to illustrate the criteria that the hospitals had satisfied within each domain. These graphs allow for the visualization of individual data points (eg, which criterion was met by a specific hospital) as opposed to visualizing the counts or proportions of hospitals that met a particular criterion, as in the case of bar charts.

### Study Population

Until the beginning of 2024, it was estimated that there were nearly 1500 public and over 300 private hospitals in Vietnam [20]. Five hospitals were purposively selected for this study based on previous interest in DHT innovation and participation in digital health research activities. Our approach aimed to sample hospitals where DHT innovation was likely to occur the soonest, rather than providing a comprehensive view of all government hospitals. Of the 5 hospitals that participated in this assessment, 4 (80%) were in Hanoi or Ho Chi Minh City, the 2 largest cities in Vietnam, and 1 (20%) was in a Mekong Delta (ie, rural) province. All are top-tier teaching public hospitals with a capacity of over 500 beds; 2 (40%) are tertiary hospitals that focus on a limited number of specialties, while 3 (60%) are general hospitals that provide care in a wide range of specialties. Most hospitals have between 8 and 12 IT staff per 1000 beds, except for 1 (20%) hospital that has an approximate ratio of over 50 per 1000 beds. Characteristics of the hospitals are shown in Table 1. To ensure confidentiality, we withheld information that could be used to identify the hospitals.

**Table 1:** Characteristics of the 5 hospitals participating in this assessment.

Hospital	Tier	Hospital type	Number of beds	Teaching hospital	IT staff:bed ratio
A	1	General hospital	>500	Yes	50:1000
B	1	Tertiary hospital	>500	Yes	13:1000
C	1	Tertiary hospital	>500	Yes	11:1000
D	1	General hospital	>500	Yes	8:1000
E	1	General hospital	>500	Yes	12:1000

### Ethical Considerations

This project was exempted from ethical review as it constituted a service review, as defined by the Oxford Tropical Research Ethics Committee (OxTREC) [21,22]. The selected hospitals were identified from a professional network that the study’s principal investigators (authors LT and CP) were part of. The hospitals were invited to participate in the assessment through an invitation letter explaining the purpose and scope of the study. Upon approval by the hospitals’ authorities, the hospital IT directors were provided with an information sheet, the questionnaire, and an instruction document. Queries regarding the assessment, if any, were clarified by the first author (DMT).

The IT directors were informed in writing that they could refuse to provide any data if they wished and that the data collected would be securely managed and only be used for the purpose of the study. Participating departments were able to review and agree on the contents of the manuscript prior to submission. No personally identifiable data were collected during the research. The research data were managed as per the Oxford University Clinical Research Unit’s data management policy. Information that can be used to identify the hospitals, such as names and the number of beds, was deidentified to ensure confidentiality. No compensation was given to the hospitals for participating in this study.

### Information Systems in Vietnamese Public Hospitals

HIS, LIS, radiology information systems (RIS), PACS, and EMRs are popular information systems in health care institutions worldwide. However, their functionalities and use cases may differ to some extent depending on the national context that they are implemented in. In this section, we provide a Vietnam-oriented description of these information systems to support the interpretation of the study’s findings.

#### Hospital Information Systems

Also known as hospital management systems, HIS are usually prioritized by Vietnamese hospitals among other information systems. They are typically used to manage outpatient services, as well as administrative and billing data emerging from all wards and departments in a hospital. HIS can be interfaced with RIS and LIS to send orders or receive imaging and laboratory results. A significant proportion of HIS in Vietnamese hospitals have been developed and provided by local vendors, such as the Corporation for Financing Promoting Technology (known as FPT), the Vietnam Posts and Telecommunications Group (known as VNPT), and Viettel [23].

#### Laboratory Information Systems

LIS allow clinical laboratories to track and use a wide range of data related to orders, specimens, results, and consumables. They reduce the turnaround time from ordering tests to collecting laboratory results through automatic processes, such as integrating with HIS to transfer orders to the laboratory and connecting with laboratory instruments to automatically receive results. Clinicians can use LIS installed in their wards or HIS interfaced with LIS to access laboratory results, when available. Many public hospitals in Vietnam have chosen LIS developed by local vendors, such as LABCONN by LABSoft (deployed in over 200 laboratories) and LABMDSOFT by MDsoft.

#### Radiology Information Systems–Picture Archiving and Communication Systems

RIS and PACS are often used together in medical imaging departments. RIS typically support managing orders, patients, and imaging procedure information. RIS can receive order information and send images and report back to HIS using HL7 messages. RIS can also send orders to imaging machines and receive processed images from PACS.

PACS comprise 4 components: imaging instruments, a secure network to transfer the images, workstations that allow doctors to view the images, and a storage system that can archive the images. PACS can thus facilitate image viewing, editing, storing, and sharing. With PACS, hospitals can eliminate physical film usage and provide multisite access to medical images through a web interface.

#### Electronic Medical Records

In Vietnam, EMR systems refer to systems implemented in hospitals, especially in inpatient wards, to manage patient care information, such as medical histories, problems, orders, tests, and medications, similar to paper charts [24]. EMRs are defined differently than electronic health records (EHRs) in Vietnam, which are defined as health records updated throughout a person’s lifetime and can cover a comprehensive range of health information, such as allergies, vaccination, family history, and outpatient visits rather than specialized care information [25]. Although the data collected by EMR systems are independently managed by hospitals, EHR data are synchronized from multiple sources, such as hospital and primary care facility visits to a centralized platform managed by the MoH [26]. In the Vietnam HIT Maturity Model, EMR systems were assessed on 4 areas as follows:

- Clinical data management functions, such as medical histories, clinical documents, and test management
- Administrative and demographics functions, including managing information of health staff and patients and managing integrations between the EMR system and other information systems in a hospital
- Medical record storage capabilities, including the required record retention duration according to health care law, record synchronization, and medical record storage and restoring
- Technical administration functions, including security, supervision, standardized terminology management, standard-based data exchange, EMR workflow management, and database backup and recovery

#### Extra Capabilities

In the Vietnam HIT Maturity Model, each digital maturity level requires several extra digital capabilities, in addition to the principal information systems, such as HIS, LIS, and EMRs (see Multimedia Appendix 1). These capabilities can be grouped as follows:

- CDRs: These are centralized data stores that gather patient data generated from other clinical information systems. Data and resources from CDRs can also be used by other systems and applications in the same network, such as standardized laboratory order vocabularies can be jointly deployed in EMRs and LIS. This assessment examined CDR implementation based on the following criteria: (1) Hospitals can establish a CDR containing standardized vocabularies, medications, orders, and laboratory tests; (2) information in CDRs can be shared with stakeholders involved in patient care; and (3) CDRs contain data on vital signs, nursing notes, and clinical procedures.
- CDSS are categorized into 3 levels: CDSS level 1 can support drug prescribing, including new prescription and represcribing; CDSS level 2 can send alerts for basic conflicts in ordering and prescribing; and CDSS level 3 can inform clinicians’ treatment plans and outcomes via appropriately customized alerts.
- Electronic ordering and inpatient order management.
- Digitizing clinical notes using electronic structured templates.
- Closed loop medication management using identification technologies such as radiofrequency identification and barcoding.
- Sharing clinical data with stakeholders involved in patient care through standardized electronic transactions, such as the HL7 Clinical Document Architecture (CDA) and the HL7 Continuity of Care Document (CCD).
- Continuous summaries of service usage data from all the departments, such as inpatient, outpatient, and emergency departments.

## Results

### Assessment Criteria

**Figure 1:**
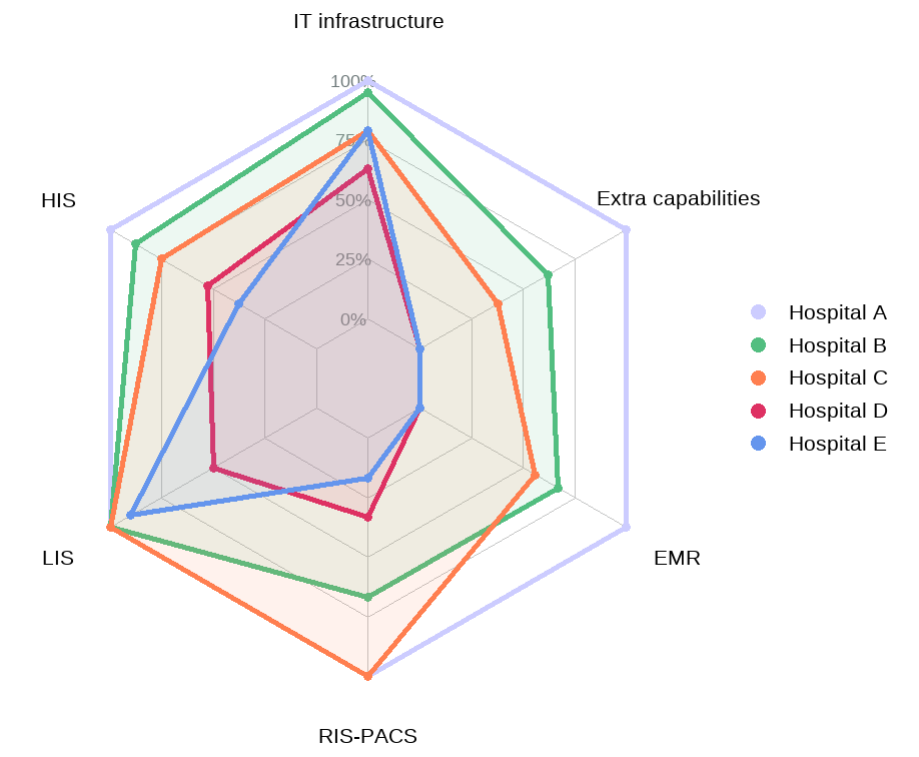
Percentage of HIT criteria satisfied in each domain across the 5 hospitals (IT infrastructure, EMR, LIS, HIS, RIS-PACS, and extra capabilities). EMR: electronic medical record; HIS: hospital information systems; HIT: health information technology; LIS: laboratory information systems; RIS-PACS: radiology information systems–picture archiving and communication systems.

Although we were not able to collect information about 3-5-year investment plans from the 5 hospitals, we were able to collect data on all the assessment criteria from the maturity model. The radar plot in Figure 1 presents the percentage of criteria the 5 hospitals met in each domain.

In general, HIS, LIS, and RIS-PACS were implemented in all 5 hospitals, albeit with varied digital maturity levels. Two hospitals were not using any EMR system or fulfilling any extra digital capability. One hospital reported meeting all the criteria across the 6 domains. In the following sections, we describe the domains in more detail and compare the implementation strategies between the hospitals.

### IT Infrastructure

**Figure 2:**
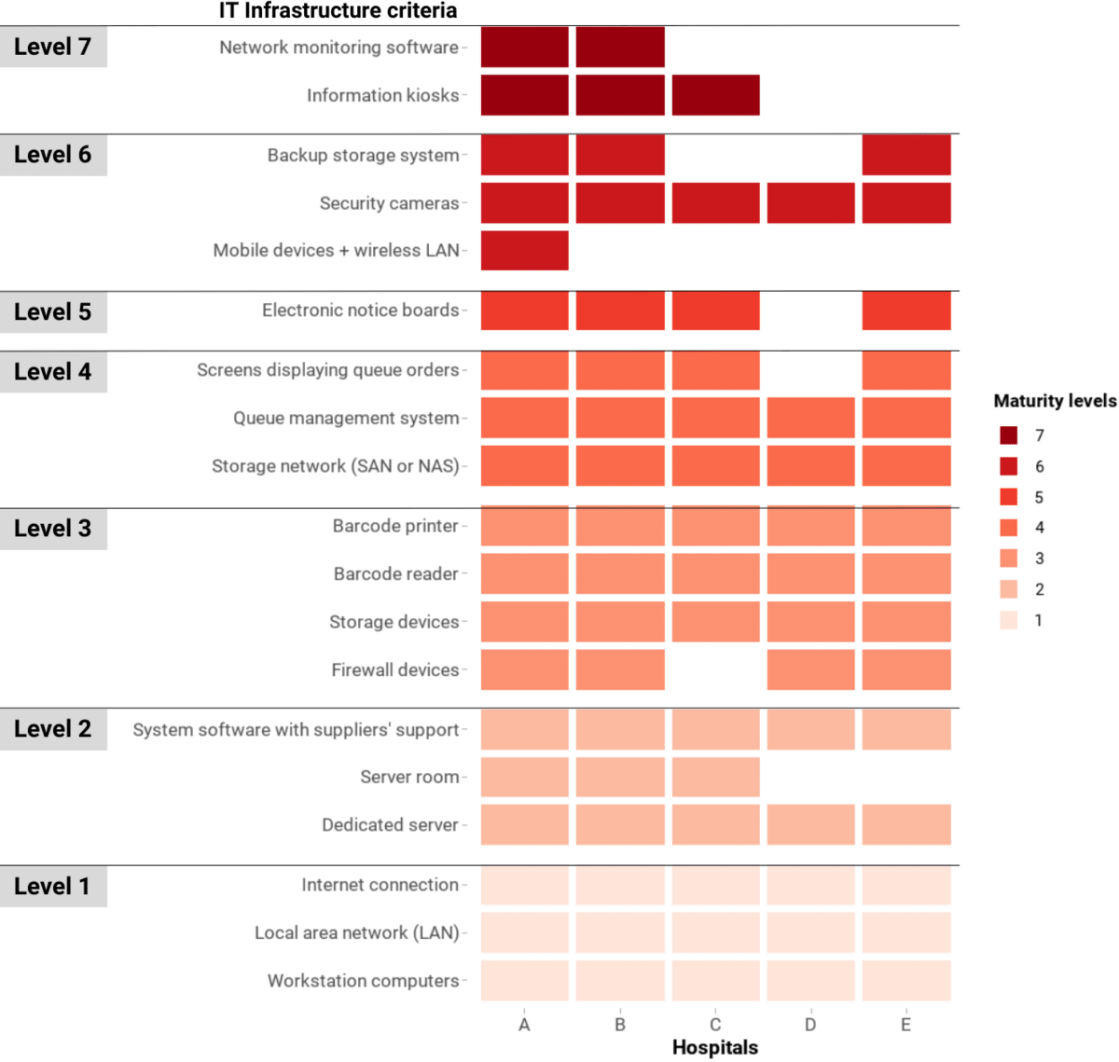
Criteria of IT infrastructure in the Vietnam HIT Maturity Model met by the 5 hospitals in this assessment, where a maximum of level 7 could be obtained. The color coding represents the maturity group that a criterion belongs to, not the entire domain’s maturity level of a hospital. Although for official purposes, a hospital needs to meet all the criteria of a lower level to progress to a higher level, this figure represents any criteria attained. HIT: health information technology; LAN: local area network; NAS: network attached storage; SAN: storage area network.

Figure 2 shows the IT Infrastructure criteria that were met by the 5 hospitals. Level 1 criteria were met by all 5 hospitals, and most hospitals reported meeting level 2-5 criteria.

All 5 hospitals had sufficient workstations for staff. They were connected to the internet and local area networks. All 5 hospitals’ digital systems and databases were run on dedicated servers, and 3 (60%) hospitals had their own on-premise server rooms with physical controls. The hospitals’ data were stored on storage devices that were supported by storage networks, such as network attached storage (NAS) or storage area network (SAN) systems. Backup storage systems were available in 3 (60%) of the 5 hospitals.

Almost all hospitals in this assessment adopted technologies to help reduce errors and improve patient flow. These included barcode readers, barcode printers, queue management systems, screens displaying queue orders, and electronic notice boards. Information kiosks were less common as only 3 (60%) of the 5 hospitals implemented this technology.

Level 6 and 7 criteria were less available in the hospitals. Only 1 (20%) hospital reported adopting mobile devices, such as tablets and smartphones, and a wireless local area network (LAN) to support hospital activities.

#### Hospital Information Systems

**Figure 3:**
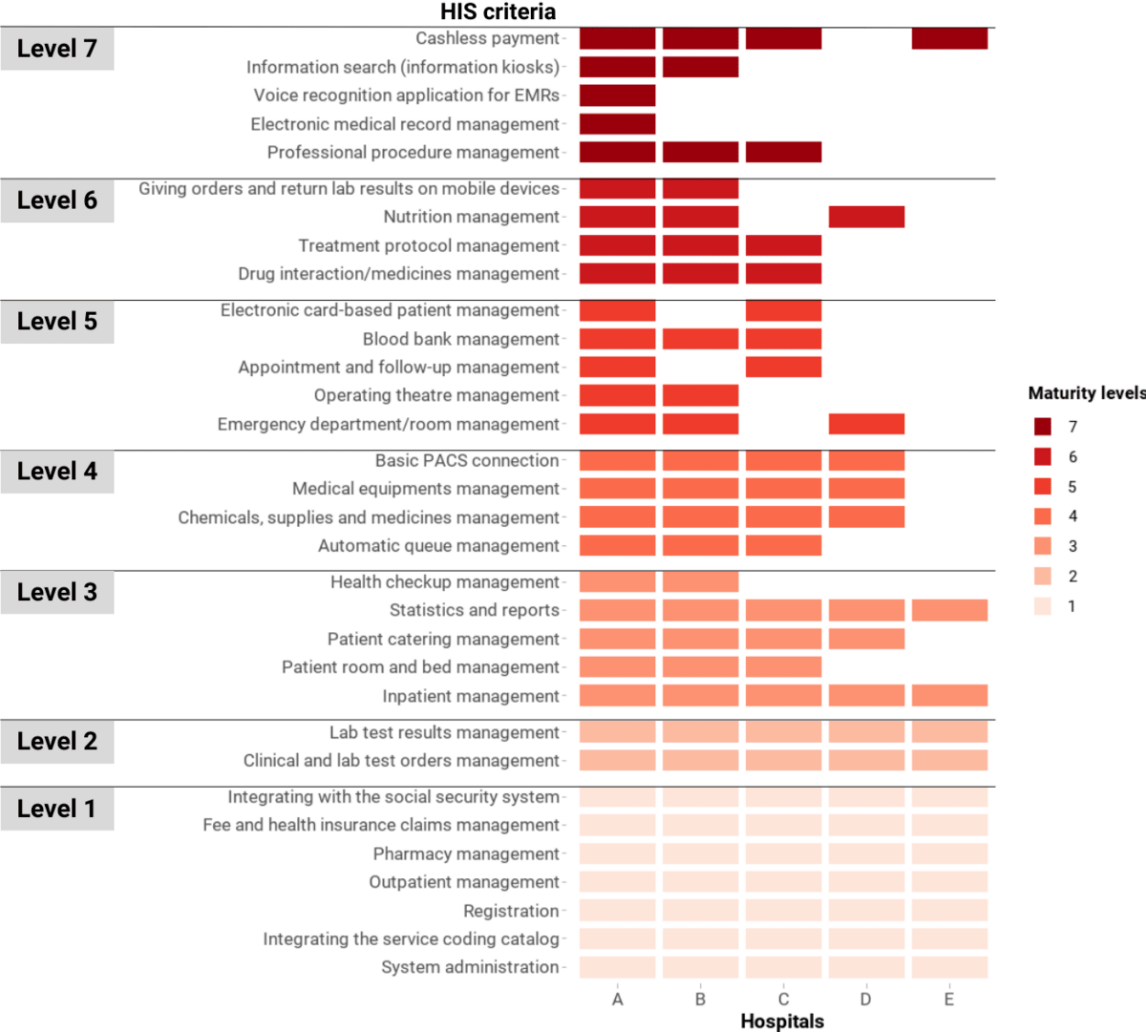
Criteria of HIS in the Vietnam HIT Maturity Model met by the 5 hospitals in this assessment, where a maximum of level 7 can be obtained. The color coding represents the maturity group that a criterion belongs to, not the entire domain’s maturity level of a hospital. Although for official purposes, a hospital needs to meet all the criteria of a lower level to progress to a higher level, this figure represents any criteria attained. EMR: electronic medical record; HIS: hospital information systems; HIT: health information technology; PACS: picture archiving and communication systems.

All 5 hospitals reported that their HIS fully met the level 1 and 2 criteria (Figure 3). These systems were able to support patient registration, outpatient services, and pharmacy stock management. Hospital fees, including social health insurance payments, were also managed in these HIS, where insurance claims were formatted into XML files following a national standard and routinely submitted to the Vietnam Social Security portal. The HIS were reportedly able to integrate Vietnam’s national coding systems to standardize the classifications of some administrative units, such as the Vietnam ethnic classification system, and clinical services, such as the WHO International Classification of Disease [27]. Medical orders and test results were assigned unique patient IDs and uploaded to the HIS. Inpatient service management and reporting functionalities (level 3) were also available in the HIS across the 5 hospitals.

Of the 5 hospitals, 4 (80%) reported that their HIS integrated with PACS and cashless payment technology. However, modules for treatment protocols, professional procedures, and drug interaction management were implemented in only 3 (60%) hospitals.

We found the functionalities that were least adopted were the ones that required integration with EMR systems (ie, voice recognition for EMRs and EMR management, available in 1, 20%, hospital). In addition, only 2 (40%) hospitals reported that their HIS could interface with information kiosks, electronic patient cards, and mobile devices.

#### Laboratory Information Systems

**Figure 4:**
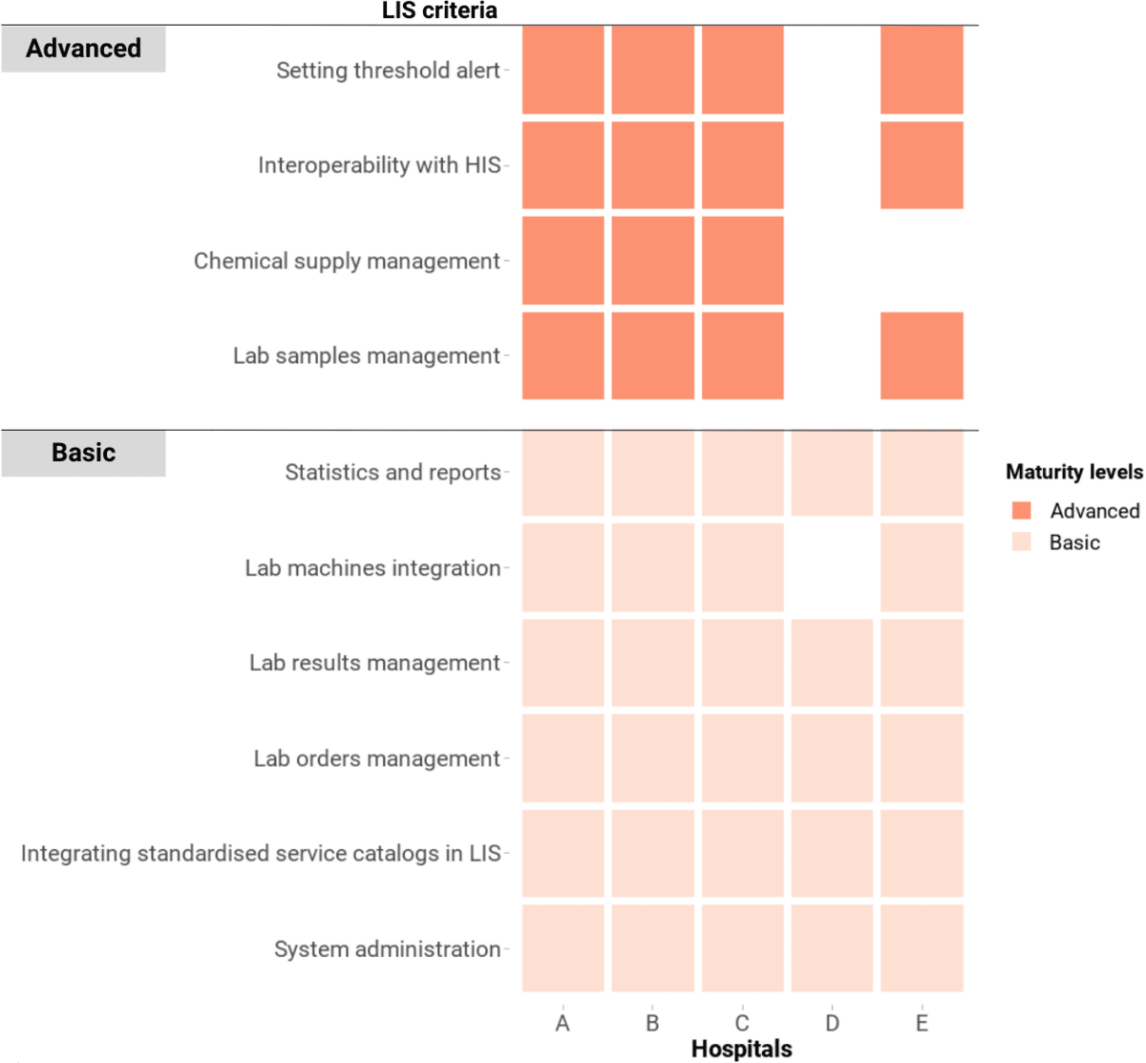
Criteria of LIS in the Vietnam HIT Maturity Model met by the 5 hospitals in this assessment, where a maximum of 2 levels can be obtained. The color coding represents the maturity group that a criterion belongs to, not the entire domain’s maturity level of a hospital. Although for official purposes a hospital needs to meet all the criteria of a lower level to progress to a higher level, this figure represents any criteria attained. HIS: hospital information systems; HIT: health information technology; LIS: laboratory information systems.

Results of our assessment showed that all 5 hospitals reported that their LIS met the basic criteria (Figure 4). All the LIS could manage hospital laboratory orders and results, as well as generate reports. Most of these systems could automatically send orders to and receive results from laboratory machines. One hospital reported that its LIS could receive results from laboratory instruments, but the orders could only be entered manually. The national dictionary system for laboratory tests was adopted across all 5 hospitals to standardize laboratory coding. The LIS of 4 (80%) hospitals satisfied all the advanced criteria, which included the ability to integrate with HIS for exchanging orders and results, set alert thresholds for laboratory results, manage chemical supply stocks, and manage laboratory specimens.

#### Radiology Information Systems—Picture Archiving and Communication Systems

**Figure 5:**
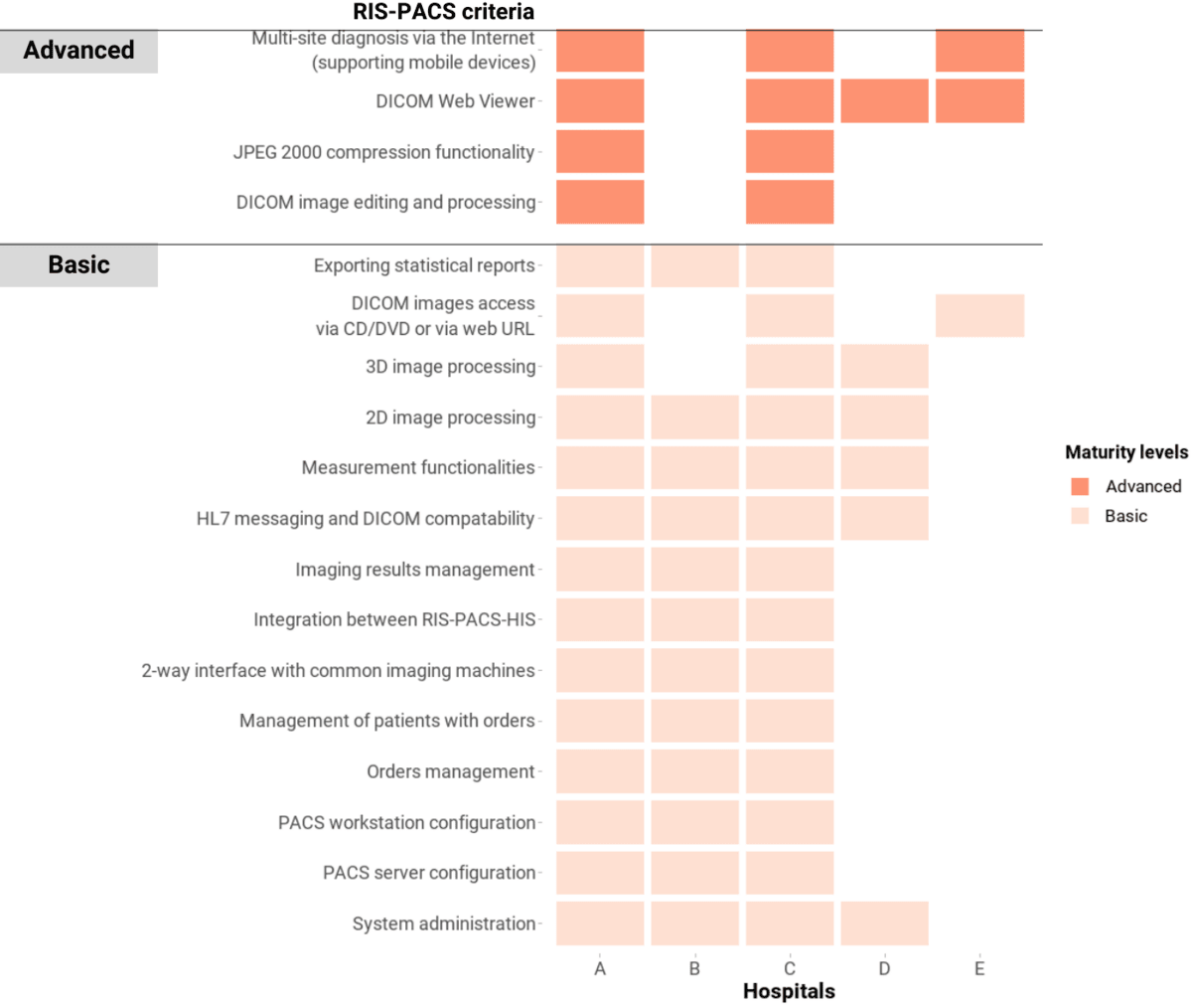
Criteria of RIS-PACS in the Vietnam HIT Maturity Model met by the 5 hospitals in this assessment, where a maximum of 2 levels can be obtained. The color coding represents the maturity group that a criterion belongs to, not the entire domain’s maturity level of a hospital. Although for official purposes a hospital needs to meet all the criteria of a lower level to progress to a higher level, this figure represents any criteria attained. DICOM: Digital Imaging and Communications in Medicine; HIS: hospital information systems; HIT: health information technology; HL7: Health Level Seven; RIS-PACS: radiology information systems–picture archiving and communication systems.

All 5 hospitals were using RIS-PACS at the time of this assessment, although with different degrees of implementation (Figure 5). Of the 5 hospitals, 2 (40%; A and C) had RIS-PACS that met all the basic and advanced criteria, 1 (20%) hospital (B) nearly satisfied all the basic and advanced criteria, and RIS-PACS from the remaining 2 (40%) hospitals (D and E) were largely operated by external vendors and only implemented a limited number of functionalities of RIS-PACS.

RIS-PACS meeting the basic criteria were able to retrieve Digital Imaging and Communications in Medicine (DICOM) images of common imaging modalities, such as X-ray, magnetic resonance imaging, and ultrasound, through 2-way interfaces. The RIS, PACS, and HIS could exchange orders and images with one another based on HL7 messaging standards. The PACS could convert DICOM images to JPEG format, and any changes to images in the PACS could be promptly updated to the HIS. Orders and radiologists’ reading result management, measurement, and reporting were also ensured by these RIS-PACS.

Hospitals meeting the advanced criteria of RIS-PACS could carry out multisite consultations through web-based access to DICOM images. One hospital reported that its PACS had adopted the HL7 FHIR standard.

#### Electronic Medical Records

**Figure 6:**
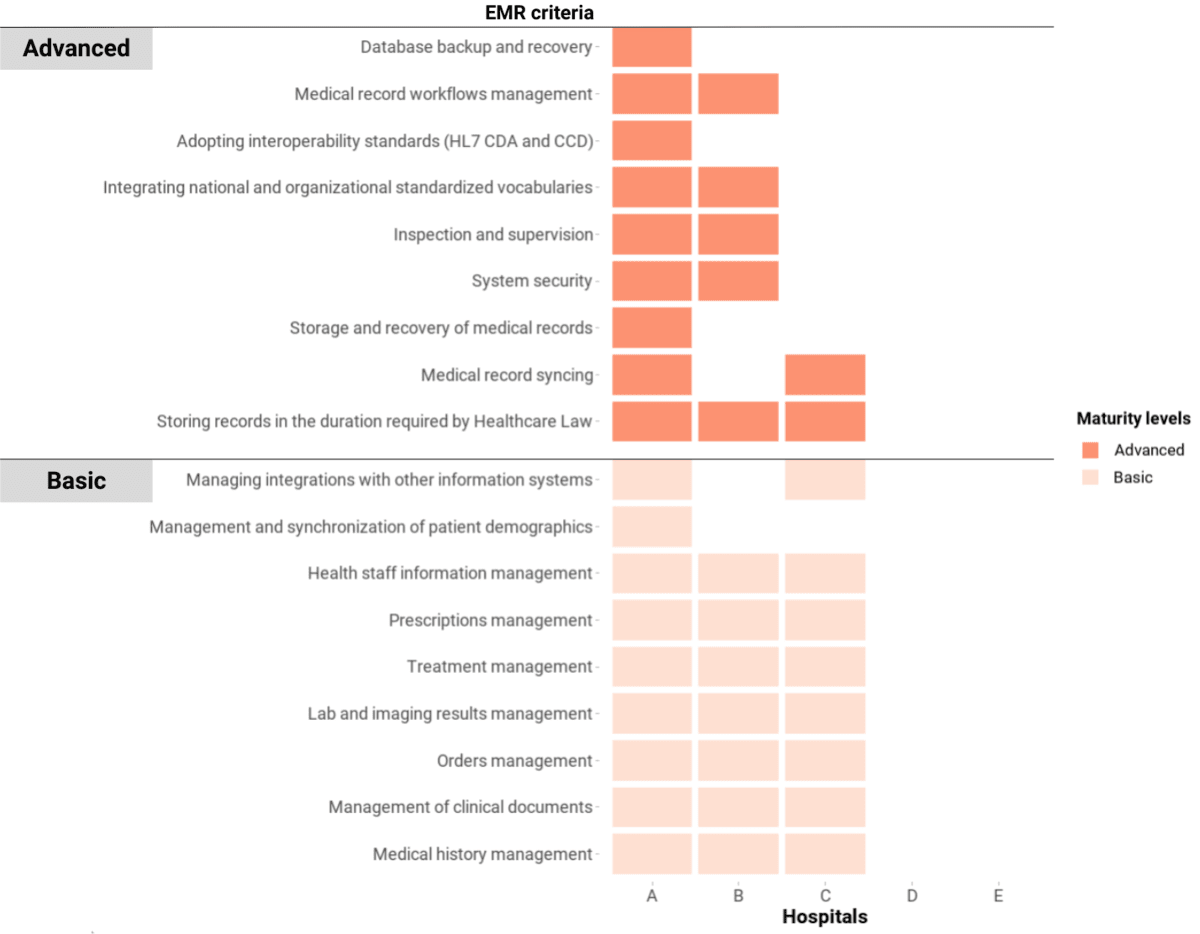
Criteria of EMRs in the Vietnam HIT Maturity Model met by the 5 hospitals in this assessment, where a maximum of 2 levels can be obtained. The color coding represents the maturity group that a criterion belongs to, not the entire domain’s maturity level of a hospital. Although for official purposes a hospital needs to meet all the criteria of a lower level to progress to a higher level, this figure represents any criteria attained. CCD: Continuity of Care Document; CDA: Clinical Document Architecture; EMR: electronic medical record; HIT: health information technology; HL7: Health Level Seven.

Generally, the EMR status was less mature than other domains (Figure 6), with only 3 (60%) hospitals reporting that they were implementing EMR systems. Only 1 (20%) of these satisfied sufficient criteria in the regulations for EMR systems [24], which allowed the hospital to completely eliminate archiving of paper records for backup and legal purposes. Nevertheless, the other EMR systems could provide clinical data management functions, including managing medical histories, clinical documents, orders, laboratory and imaging results, treatments, and prescriptions.

An EMR system fully compliant with the regulations for EMRs could ensure storage capacity, backup, and recovery capabilities. Interoperability standards, such as the HL7 CDA, the HL7 CCD, and HL7 FHIR, were implemented. Information security measures, such as authentication, audit trail, and encryption, were in place. Digital signatures were used to authenticate medical records and orders.

#### Extra Capabilities

**Figure 7:**
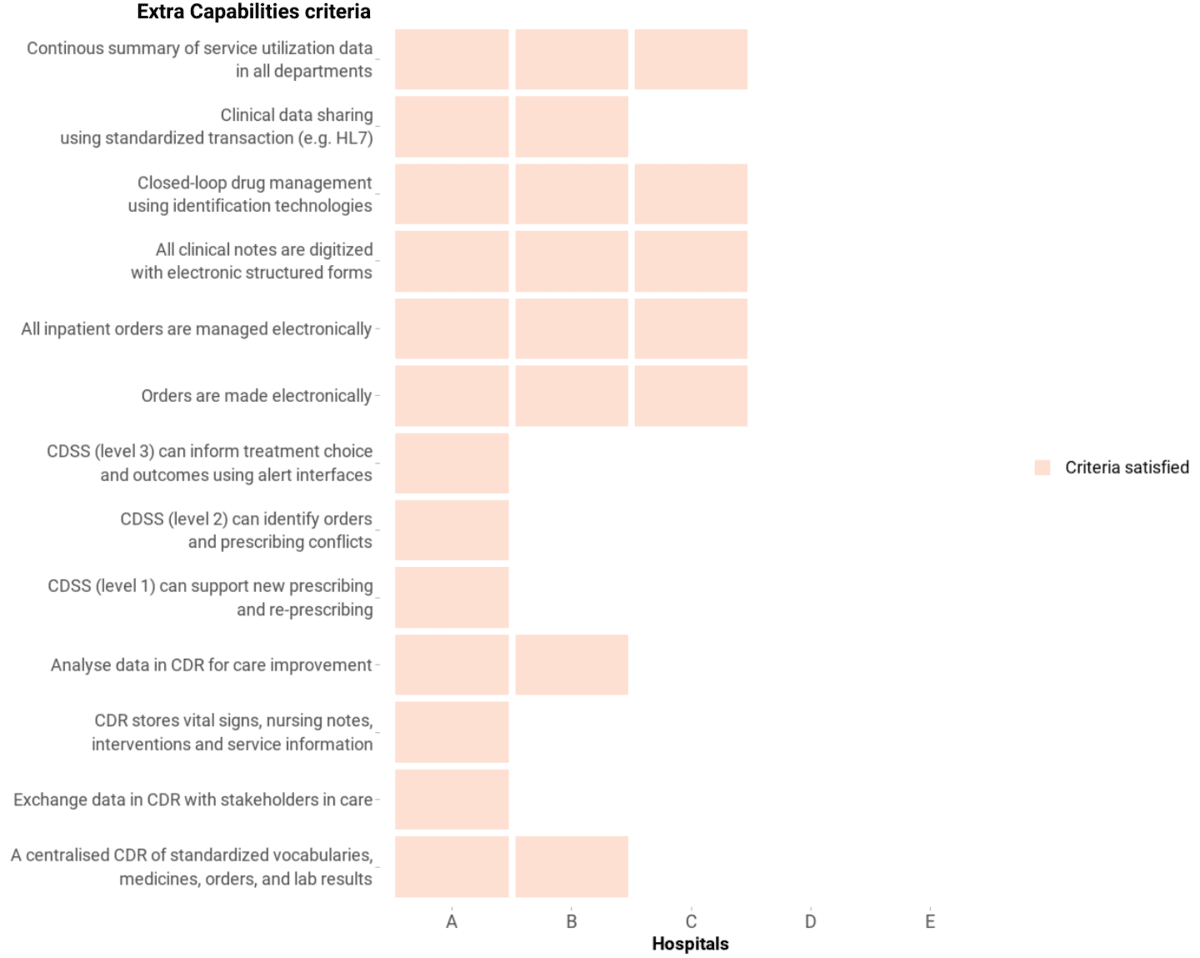
Extra capability criteria in the Vietnam HIT Maturity Model met by the 5 hospitals in this assessment. CDR: clinical data repository; CDSS: clinical decision support systems; HIT: health information technology; HL7: Health Level Seven.

The availability of these capabilities in the 5 hospitals is shown in Figure 7. Notably, 1 (20%) hospital reported achieving all the extra capabilities, with 2 (40%) hospitals confident about sharing clinical data using HL7 standards (although they were not currently sharing data at the time of the study). The adoption of CDRs and CDSS to support clinical tasks, such as giving orders, prescribing, and clinical decision-making, was only available in 1 (20%) hospital. However, all the hospitals with an EMR system reported being able to digitize all clinical documents and allow clinicians to give orders in the electronic environment.

## Discussion

### Principal Findings

This study assessed the implementation of some key digital health systems in 5 top-tier hospitals in Vietnam. Overall, many processes related to administration, laboratory tests, and medical imaging were digitized in the hospitals through the implementation of HIS, LIS, and RIS-PACS on the basis of adequate basic IT infrastructures. The digital maturity of these systems, however, varied significantly between the hospitals, although all are large top-tier hospitals. EMR systems were implemented in 3 of the 5 hospitals. Although these systems could satisfy a wide range of data collection needs, their integration with CDRs and CDSS was sparse.

### Comparison With Prior Works

This is the first original research that examined digital health system implementation in Vietnamese hospitals since the Vietnam MoH enacted the National EMR plan and the Vietnam HIT Maturity Model in 2018 as part of the sectorwide digital transformation agenda [11,21,24]. Academic publications on adoption and implementation of hospital-based digital health systems are limited [2]. Most of the previous studies have been conducted before 2014 and focused on the readiness to transition from paper-based processes to electronic systems rather than describing DHT adoption [28,29]. Other publications have described the development and pilots of technical solutions, such as LIS, data retrieval from medical devices, and data visualization for EMRs. We could not find any research exploring the digital systems currently used in hospitals in Vietnam [30-33].

Muinga et al [34] showed that most of the digital health systems adopted in Kenyan public hospitals from 2014 to 2016 were aimed at administrative and billing purposes rather than supporting clinical tasks. Systems such as LIS, RIS, and PACS, when available, were usually stand-alone systems and lacked interoperability with other systems in the same institution [34]. Our study found that all the Vietnamese hospitals assessed had gone beyond administrative systems and had used clinical information systems that are interoperable with each other, especially with HIS. EMRs, as a crucial clinical information system, had been implemented in 3 of the 5 hospitals to capture a wide range of data, such as medical histories, clinical documents, orders, and test results. However, the adoption of EMR capabilities, such as CDRs and CDSS, was limited, even though these are large teaching hospitals at the top tier of the Vietnamese health system. This is inferior to the digital maturity status of public hospitals in Turkey, as over 98% of the Turkish hospitals surveyed reported having CDRs regardless of their size [35]. In addition, CDSS for medication orders and nonmedication orders were implemented in 71% and over 57% of Turkish public hospitals, respectively. The early use of EMRAM to guide the national digital health care transformation may be an important facilitator for this wide adoption of CDRs and CDSS in Turkish hospitals [36].

The Vietnam HIT Maturity Model [11] that this study’s questionnaire was based on and the HIMSS EMRAM [9] share several similarities regarding assessment criteria. For example, CDRs, CDSS, closed-loop medication administration, and electronic documentation are included in both models, including the maturity stage that these systems belong to. However, EMRAM particularly focuses on EMR capabilities, in which systems such as laboratory, radiology, and pharmacy are seen as EMR ancillaries, while CDRs and CDSS are crucial components deciding the maturity of the EMR system. All the ancillary systems need to be implemented as early as in stage 1 of EMRAM. The Vietnam HIT Maturity Model, in contrast, splits the ancillaries into basic and more advanced maturity levels along the digital maturity roadmap. Systems such as CDRs and CDSS are the extra capabilities that should be met, alongside other domains, such as HIS, LIS, RIS, and PACS, so that a hospital can move up to a higher digital maturity level. Although the division of digital systems into smaller milestones may allow hospitals to better benchmark their digital maturity and plan for investments, the requirement to meet digital maturity criteria across multiple domains can be challenging for hospitals in a resource-limited setting such as Vietnam. Duncan et al [10] discussed that most published maturity models, including EMRAM, are not assessed in LMICs, questioning their applicability in cultures different from those of high-income countries, such as the United States and the United Kingdom. In addition, the implementation of EMRAM poses the risk of investing in complex systems without meeting the organization’s local needs [37].

### Integrating New DHTs Into the Existing System

Most hospitals participating in this assessment had a low adoption rate of EMRs, CDSS, and CDRs. The low number of complete EMR systems may be caused by the cost required to upgrade the infrastructure and a lack of the required technical capabilities to meet all the requirements in EMR regulations. It is difficult for hospitals with incomplete EMR systems and CDRs to embark on using CDSS. Integrating, implementing, and maintaining CDSS is usually complex and depends on multiple factors, such as the availability and interoperability of data, especially from EMRs; an updated knowledge base; and understanding of the hospital’s workflow [38].

The wide availability of HIS, LIS, and RIS-PACS in the hospitals participating in our study suggests that digital systems for administrative and billing purposes and simpler systems to implement tend to be prioritized. Many hospitals may choose to implement clinical documentation functions before upgrading the other components to fully comply with EMR regulation. This leads to an increasing amount of medical data that are electronically available and useful for AI training and implementation even without full EMR implementation. However, the scarcity of CDSS, CDRs, and standard-based data-sharing experience found in this assessment suggests challenges that stakeholders may face when seeking to integrate AI-enabled solutions in Vietnamese hospitals. Although these challenges should be explored in further research, we believe a lack of national frameworks and detailed guidance for meaningful and secure clinical data exchange is 1 of the key barriers. In addition, recently, Chanh et al [39] mentioned exploring system interoperability, collaboration with local stakeholders from multiple disciplines, and understanding local data-sharing policies as some of the key considerations for applying AI in Vietnam.

### Strengths and Limitations

We examined the adoption of digital health systems using the criteria published in the Vietnam HIT Maturity Model. This is a national benchmarking tool for HIT implementation in Vietnamese public hospitals, making it familiar to the hospitals participating in this study.

Only 5 public hospitals at the top-tier level were included in this assessment, so our findings do not represent the overall picture of HIT implementation in Vietnamese hospitals. The hospitals participating in this study were ones that had previous interest in DHT implementation and digital health innovation research. However, it is likely that hospitals at low tiers and located in less-funded regions have lower digital maturity across all the domains, especially in the EMR and extra capabilities domains.

### Implications for Further Study

The adoption of digital health systems in this study was assessed based on technological criteria, such as the software functions that were available. Future work could address other dimensions related to the implementation of these systems, such as the organizational context and human factors [40]. The use of qualitative methods can be particularly suitable for such research questions as they can unveil the characteristics unique to technology adoption in hospitals in LMIC settings, such as Vietnam.

### Conclusion

Several major public hospitals in Vietnam have a sound digital infrastructure in place to support fundamental administrative and clinical tasks, such as patient management, insurance claims, laboratory result management, and medical imaging inspection. Most of the hospitals have implemented EMR systems to a basic level that prioritizes data collection, management, and access. Nevertheless, the more advanced level of data management and use via CDRs and CDSS is not common. This can be seen as a barrier to the introduction of new DHTs in these hospitals. Along with the increased amount of data collected by the systems, the adoption of HL7, DICOM, and other international standards can be seen as a facilitator for new DHTs, such as AI-based CDSS, to be implemented in these hospitals.

## Appendix

Multimedia Appendix 1 Vietnam HIT Maturity Model issued in 2017 by the Vietnam MoH. HIT: health information technology; MoH: Ministry of Health.

Multimedia Appendix 2 The hospital health information systems assessment questionnaire.

## Abbreviations

AI: artificial intelligence
CCD: Continuity of Care Document
CDA: Clinical Document Architecture
CDR: clinical data repository
CDSS: clinical decision support systems
DHT: digital health technology
DICOM: Digital Imaging and Communications in Medicine
EMR: electronic medical record
EMRAM: Electronic Medical Record Adoption Model
FHIR: Fast Healthcare Interoperability Resources
HIMSS: Healthcare Information and Management Systems Society
HIS: hospital information systems
HIT: health information technology
HL7: Health Level Seven
LAN: local area network
LIS: laboratory information systems
LMIC: low- and middle-income country
MoH: Ministry of Health
NAS: network attached storage
PACS: picture archiving and communication systems
RIS: radiology information systems
RIS-PACS: radiology information systems–picture archiving and communication systems
SAN: storage area network
SDG: Sustainable Development Goal
WHO: World Health Organization

## Data Availability

The data set generated and analyzed during this study is available upon request through the Oxford University Clinical Research Unit’s managed data access scheme [41].
