# Supplementary material for: Status of Digital Health Technology Adoption in 5 Vietnamese Hospitals: Cross-Sectional Assessment
Source: JMIR Form Res. 2025 Feb 6;9:e53483. doi: 10.2196/53483 (PMC11843058; doi:10.2196/53483)
Supplement: Multimedia Appendix 1 [file formative_v9i1e53483_app1.docx]

**Multimedia Appendix** **1.** The Vietnam HIT^a^ Maturity Model issued in 2017 by the Vietnam MoH^b^ [1].

| HIT | IT infrastructure | HIS^c^ | LIS^d^ | RIS-PACS^e^ | EMR^f^ | Administration and operation software | Security and information safety | Nonfunctional criteria | Extra capabilities |
| --- | --- | --- | --- | --- | --- | --- | --- | --- | --- |
| Level 7 | Level 7 | Level 7 | Advanced | Advanced | Advanced | Advanced | Advanced | Advanced | - Paperless hospital if all relevant criteria are met - CDSS^g^ level 3 supporting doctors’ decisions related to treatment protocols and treatment results using suitably customized templates - Data in CDRs^h^ analyzed to improve care quality, patient safety, and care efficiency - Clinical data readily shared for stakeholders in care coordination based on HL7^i^ standards - Continuous reports of hospital services using the data collected |
|  |  |  |  |  |  |  |  |  |  |
|  |  |  |  |  |  |  |  |  |  |
|  |  |  |  |  |  |  |  |  |  |
| Level 6 (smart hospital) | Level 6 | Level 6 | Advanced | Advanced | Basic | Advanced | Advanced | Advanced | - CDSS level 2 providing: evidence-based warnings for treatment (ie, health and medicine advice, drug information/interaction check, and initial order/prescription violation identification rules) - All structured forms (ie, progress notes, consultation notes, problem lists, discharge summaries) digitalized - Closed-loop management of drugs using identification technologies to assist drug administration |
|  |  |  |  |  |  |  |  |  |  |
|  |  |  |  |  |  |  |  |  |  |
| Level 5 | Level 5 | Level 5 | Advanced | Advanced | N/A^j^ | Basic | Basic | Basic | - PACS replacing physical films |
| Level 4 | Level 4 | Level 4 | Advanced | Basic | N/A | Basic | Basic | Basic | - PACS allowing doctors to access images outside the imaging department - Electronic ordering - Electronic management of inpatient orders |
|  |  |  |  |  |  |  |  |  |  |
| Level 3 | Level 3 | Level 3 | Basic | N/A | N/A | Basic | Basic | Basic | - Electronic records having digital vital sign records, nursing notes, medical procedures, and surgical procedures stored in CDRs - CDSS level 1 assisting electronic prescription (new or historic prescription) - Pharmacy information available in the hospital network and supported with CDSS |
|  |  |  |  |  |  |  |  |  |  |
|  |  |  |  |  |  |  |  |  |  |
| Level 2 | Level 2 | Level 2 | N/A | N/A | N/A | N/A | N/A | N/A | - A CDR consisting nomenclature and coding systems, pharmacy, orders, and test results (if available) - Data in CDRs shared between stakeholders for care coordination |
|  |  |  |  |  |  |  |  |  |  |
| Level 1 | Level 1 | Level 1 | N/A | N/A | N/A | N/A | N/A | N/A | - Patient information accessible electronically |

^a^HIT: health information technology.

^b^MoH: Ministry of Health.

^c^HIS: hospital information systems.

^d^LIS: laboratory information systems.

^e^RIS-PACS: radiology information systems–picture archiving and communication systems.

^f^EMR: electronic medical record.

^g^CDSS: clinical decision support systems.

^h^CDR: clinical data repository.

^i^HL7: Health Level Seven.

^j^N/A: not applicable.

## Reference

1. Thông tư 54/2017/TT-BYT về Bộ tiêu chí ứng dụng công nghệ thông tin tại các cơ sở khám bệnh, chữa bệnh. Vietnam Ministry of Health. 2017. URL: https://thuvienphapluat.vn/van-ban/Cong-nghe-thong-tin/
