# Supplementary material for: Status of Digital Health Technology Adoption in 5 Vietnamese Hospitals: Cross-Sectional Assessment
Source: JMIR Form Res. 2025 Feb 6;9:e53483. doi: 10.2196/53483 (PMC11843058; doi:10.2196/53483)
Supplement: Multimedia Appendix 2 [file formative_v9i1e53483_app2.docx]

Multimedia Appendix 1.

The Hospital Health Information Systems Questionnaire

The heads of IT departments answer ‘satisfied’ or ‘not satisfied’ for each of the following criteria.

# IT infrastructure

1. Workstation computers can satisfy the minimum requirements for implementing health IT
2. Local area network
3. Internet connection
4. Dedicated server (application or database)
5. Server room (equiped with fire safety equipments, temperature and humidity monitoring equipments, entry control equipments)
6. System software (operating system, database management system) with supports from providers (except for open-source softwares)
7. Firewall device
8. Storage devices (storage servers or external storage devices)
9. Barcode reader
10. Barcode printer
11. Storage network (SAN or NAS)
12. Queue management system
13. Screens displaying queue orders
14. Electronic notice boards (hospital news, health service prices, etc.)
15. Mobile devices (tablets, smartphones), wireless LAN
16. Security cameras
17. Backup storage systems
18. Information kiosks (provide information about hospital and medical examination and treatment for patients and their family)
19. Network monitoring software

# Hospital Information System

1. System administration (User management, configuration management)
2. Integrating the service coding catalog in HIS
3. Registration
4. Outpatient management
5. Pharmacy management
6. Hospital fee and health insurance reimbursement management
7. Connection to VSS system for health insurance reimbursement (using XML)
8. Clinical and lab order management
9. Lab result management
10. Inpatient management
11. Rooms and beds management
12. Catering management
13. Reports making
14. Health checkup management
15. Automatic queue management
16. Chemicals, supplies and pharmacy management
17. Medical equipments management
18. Basic PACS connection
19. Emergency department/room management
20. Operating theatre management
21. Appointment and follow-up management
22. Blood bank management (if applicable)
23. Electronic card-based patient management
24. Drug interaction/medicines management
25. Treatment protocol management
26. Providing access to presciptions, orders and test results on mobile devices
27. Professional procedures management
28. Electronic medical records management
29. Voice recognition for EMRs
30. Information browsing (information kiosks)
31. Cashless payment

Radiology Information System - Picture Archiving and Communication System

1. System administration
2. PACS server configuration
3. PACS workstation configuration
4. Orders management
5. Patients receiving orders management
6. 2-way interface with common imaging devices (CT, MRI, Radiography, DSA, ultrasound)
7. Integrating with HIS:
   - RIS receives orders from HIS. RIS then transmits the order to the imaging device utilizing HL7.
   - PACS receives processed images from doctor's workstations.
   - PACS converts DICOM images to JPEG and transfers them to RIS. RIS then transfers the images to HIS to complete medical records.
   - 2-way interoperability between PACS and HIS (any changes made in PACS would be reflected in HIS and vice versa)
8. Imaging results management
9. HL7 messaging and DICOM standard compatibility
10. Measuring functionalities
11. 2D image processing
12. 3D image processing
13. Exporting DICOM images and a DICOM viewer software to CD/DVD or providing web-based viewing.
14. Reports making
15. DICOM image editing and processing
16. JPEG 2000 compression functionality
17. DICOM Web Viewer
18. Multi-site diagnosis via Internet (accessible via mobile devices such as smartphones and tablets)

# Electronic Medical Record

1. Medical history management
2. Clinical documents management
3. Orders management
4. Lab results management
5. Treatment management
6. Prescription management
7. Healthcare workers information management
8. Patients demographics management, including synchronization of demographics
9. Integrations management
10. Capability to archive medical records under the required duration in Healthcare Law
11. Medical records synchronization
12. Saving and recovering medical records
13. System security
14. Inspection and supervision
15. Internal and standard common service list management
16. Interoperability based on standards (exchanging electronic medical records with HL7 CDA, CCD)
17. Workflow and rules management on EMR system
18. Database backup and recovery

# Laboratory Information System

1. System administration
2. Nonmenclature and Coding Systems management
3. Lab orders management
4. Lab results management
5. Lab machine integration (issue an order and receive laboratory test results from the machines)
6. Reports making
7. Lab samples management
8. Chemicals management
9. Interoperability with HIS (receiving requests from HIS and synchronizing lab test results with HIS)
10. Setting threshold alert

# Extra capabilities

1. Developed a centralized CDR consisting of nomenclature and coding catalogs, pharmacy, orders, and lab results (if applicable)
2. Exchange data stored in the CDR to stakeholders involved in patient care
3. Digital records including vital signs (hear rate, temperature, and blood pressure), nursing notes, and information about services and interventions are stored in the CDR
4. Analyse data in the CDR to provide insights for improving patient care, patient safety, and efficiency.
5. The CDSS can support electronic drug prescribing, including creating new prescription and re-prescribing
6. The CDSS can identify conflicts in orders and drug prescribing using evidence-based rules
7. The CDSS can support doctors decisions related to treatment regimens and treatment outcomes via suitable alert interfaces
8. Doctors give orders in the electronic environment
9. The system can manage all doctors orders from the inpatient department
10. Digitalise all structured clinical notes used by doctors and nurses, including progress notes, consultation notes, problem lists, and discharge summaries.
11. Closed-loop management of drugs, using identification technologies such as RFID to assist drug administration.
12. Clinical data is ready for sharing between the stakeholders involved in patient care with HL7 standards
13. Generate frequent summary reports for hospital deparments (inpatient, outpatient, emergency, clinics, etc.)
